# Cornelian Cherry Iridoid-Polyphenolic Extract Improves Mucosal Epithelial Barrier Integrity in Rat Experimental Colitis and Exerts Antimicrobial and Antiadhesive Activities *In Vitro*

**DOI:** 10.1155/2020/7697851

**Published:** 2020-11-20

**Authors:** Marta Szandruk-Bender, Maria Rutkowska, Anna Merwid-Ląd, Benita Wiatrak, Adam Szeląg, Stanisław Dzimira, Beata Sobieszczańska, Małgorzata Krzystek-Korpacka, Alicja Z. Kucharska, Agnieszka Matuszewska, Beata Nowak, Narcyz Piórecki, Anna Duda-Madej, Urszula Walczuk, Michał Turniak, Iwona Bednarz-Misa, Tomasz Sozański

**Affiliations:** ^1^Department of Pharmacology, Wroclaw Medical University, Mikulicza-Radeckiego 2, 50-345 Wrocław, Poland; ^2^Department of Pathology, Wroclaw University of Environmental and Life Sciences, Norwida 31, 50-375 Wrocław, Poland; ^3^Department of Microbiology, Wroclaw Medical University, Chałubińskiego 4, 50-368 Wrocław, Poland; ^4^Department of Medical Biochemistry, Wroclaw Medical University, Chałubińskiego 10, 50-368 Wrocław, Poland; ^5^Department of Fruit, Vegetable and Plant Nutraceutical Technology, Wroclaw University of Environmental and Life Sciences, Chełmońskiego 37, 51-630 Wrocław, Poland; ^6^Bolestraszyce Arboretum and Institute of Physiography, 37-700 Przemyśl, Poland; ^7^Department of Tourism and Recreation, University of Rzeszow, Towarnickiego 3, 35-959 Rzeszów, Poland

## Abstract

**Background and Aims:**

Inflammatory bowel disease pharmacotherapy, despite substantial progress, is still not satisfactory for both patients and clinicians. In view of the chronic and relapsing disease course and not always effective treatment with adverse effects, attempts to search for new, more efficient, and safer substances are essential and reasonable. This study was designed to elucidate the impact of cornelian cherry iridoid-polyphenolic extract (CE) and loganic acid (LA) on adherent-invasive *E*. *coli* growth and adhesion *in vitro* and to assess the effect of pretreatment with CE or LA on the course of intestinal inflammation in rat experimental colitis compared with sulfasalazine.

**Methods:**

Antibacterial and antiadhesive activities of CE and LA were assessed using microdilution, Int407 cell adherence, and yeast agglutination assays. The colitis model was induced by 2,4,6-trinitrobenzenesulfonic acid. Studied substances were administered intragastrically for 16 days prior to colitis induction. Body weight loss; colon index; histological injuries; IL-23, IL-17, TNF-*α*, and chemerin levels; and STAT3, Muc2, and TFF3 mRNA expression were evaluated.

**Results:**

Only CE exerted antimicrobial and antiadhesive activities *in vitro* and alleviated colonic symptoms. CE coadministrated with sulfasalazine was more effective than single compounds in reversing increased concentrations of TNF-*α*, IL-17, and chemerin and decreased Muc2 mRNA expression.

**Conclusions:**

CE exerted a protective effect against experimental colitis via impaired mucosal epithelial barrier restoration and intestinal inflammatory response attenuation and given concomitantly with sulfasalazine counteracted colitis in a more effective way than sulfasalazine alone, which indicates their synergistic interaction. The beneficial effect of CE may also be due to its bacteriostatic and antiadhesive activities.

## 1. Introduction

Inflammatory bowel disease (IBD), a group of chronic and recurring intestinal disorders which comprises two main entities, Crohn's disease (CD) and ulcerative colitis (UC), has become a global healthcare problem. IBD affects at least 0.5% of the population in westernized countries with accelerating incidence in newly industrialized regions [1]. IBD decreases patients' quality of life, has a poor prognosis, and leads to lifelong morbidity. Both forms of IBD are differentiated by their location and extent of inflammatory changes in the gastrointestinal tract. CD is characterized by transmural inflammation that may appear in any part of the gastrointestinal tract, whereas UC by inflammation of the colonic mucosa and submucosa. Initially, both CD and UC are associated with low-grade fever, fatigue, bloody diarrhea with mucorrhea, abdominal pain, unintended weight loss with reduced appetite, and anemia. Symptoms range from mild to severe during relapses and may disappear during remissions. The differences between Crohn's disease and ulcerative colitis patients become more evident with the progression of the disease, along with the development of intestinal and extraintestinal complications [2, 3].

The altered immune response is an indisputable factor relevant to the pathogenesis of IBD regardless of the controversy over the type and origin of the antigen. It is presumed that a complex interaction between heritable traits and environmental and microbial factors may lead to enhanced immune response. Even though the etiopathogenesis of IBD remains not completely understood, there is growing evidence that increased infiltration, proliferation, and activation of Th17 cells and increased concentrations of IL-23/Th17 pathway proinflammatory cytokines including, inter alia, tumor necrosis factor *α* (TNF-*α*), interleukin 23 (IL-23), interleukin 17 (IL-17), and chemerin play a pivotal role in the development and maintenance of mucosal inflammation and lead to gut tissue damage. It is noteworthy that Th17 cells are firmly dependent on transcription factor STAT3 activation [2, 4].

Inappropriate immune response and intestinal inflammation might be triggered by mucosal epithelial barrier disorders, such as impaired function and reduced number of goblet cells, abnormal mucus production, and decreased mucus layer thickness. The deficiency of mucins and trefoil peptides, especially mucin 2 (Muc2) and trefoil factor-3 (TFF3), secreted by goblet cells, makes intestinal tissues vulnerable to inflammation and impedes mucosal repair and restitution [5]. The reciprocal regulation among the intestinal epithelial cells (IECs), a variety of immune cells, and microbiota is critical for maintaining the integrity of the mucosal barrier. In IBD patients, gut microbiota differs qualitatively and quantitatively compared to that of healthy people [1]. Intestinal microbiome dysbiosis is characterized by an increase in the number of mucosa-associated bacteria (e.g., an enteropathogenic strain of the B2 phylotype *E*. *coli* termed AIEC) and a reduction in the overall microbial diversity. AIEC adheres to and invades IECs and can survive and replicate within macrophages, inducing TNF-*α* secretion and promoting granuloma formation [6, 7]. Regardless of whether the change in the microbiome functioning is the cause or effect of IBD, it is plausible that clinical remission is not accompanied by a restoration in the gut microbial balance, which may lead to future relapses influencing the severity of the disease [8].

Despite meaningful progress in IBD therapy, the current treatment has substantial limitations with regard to safety and efficacy. Aminosalicylates and glucocorticosteroids are the drugs of choice for the treatment of mild to moderate IBD, while immunosuppressants and biological agents are reserved for more severe cases or nonresponsive patients. Such treatment may cause serious adverse effects, especially during long-term administration, and relapse upon drug discontinuation [9, 10]. Thus, there is still an unmet clinical need for the identification of new, more efficient, and safer therapies for IBD. A tempting approach in future therapies might be reinforcement of the mucosal epithelial barrier to achieve long-term remission. The improvement of gut barrier integrity alone might not be sufficient in severe inflammatory diseases but in combination with standard therapy might have additional benefits [11]. Similarly, targeting AIEC colonization via antiadhesive compounds might be another attractive adjuvant strategy to the prevention and treatment of IBD [12].

One of the approaches for the development of future IBD treatments or adjuvant therapy is the appraisal of plant-derived natural compounds that target various inflammation-related molecules and signaling pathways associated with IBD. Several studies based on *in vitro* and *in vivo* assays revealed antioxidant, anti-inflammatory, immunomodulatory, antimicrobial, and analgesic activities of cornelian cherry iridoid-polyphenolic extract (CE) and its compounds, especially loganic acid (LA). The bulk of research indicated that the beneficial effects of cornelian cherry fruits on various physiological parameters are due to the presence of polyphenols and iridoids [13–15].

The current study was undertaken to elucidate the impact of cornelian cherry iridoid-polyphenolic extract and loganic acid on pathogenic *E*. *coli* strain LF82 growth and adhesion to intestinal epithelial cells *in vitro* as well as assessing the effect of pretreatment with CE or LA on the course of intestinal inflammation in 2,4,6-trinitrobenzenesulfonic acid (TNBS) experimental colitis in rats. Moreover, this study is aimed at comparing the action of CE and LA with that of sulfasalazine, a well-established drug in IBD, and at elucidating whether CE or LA concomitantly administrated with sulfasalazine might act synergistically with sulfasalazine.

## 2. Materials and Methods

### 2.1. Plant Material

#### 2.1.1. Sample Preparation of Cornelian Cherry Iridoid-Polyphenolic Extract and Loganic Acid

Cornelian cherry (*Cornus mas* L.) fruits were collected in the Bolestraszyce Arboretum and Institute of Physiography, Poland. The voucher specimen (BDPA 3967) has been deposited at the Herbarium of the Arboretum in Bolestraszyce, Poland. The investigated cornelian cherry iridoid-polyphenolic extract and loganic acid were prepared from cornelian cherry fruits by the Department of Fruit, Vegetable and Plant Nutraceutical Technology at the Wroclaw University of Environmental and Life Sciences according to the method described by Kucharska et al. and Sozański et al. [16, 17]. CE was obtained after purification on XAD-16 Amberlite resin (Rohm and Haas, France) in a column and then concentrated using a Rotavapor (Unipan, Poland) and lyophilized (Alpha 1-4 LSC, Germany) [16]. LA was fractionated from CE by a polyamide (Macherey-Nagel-CC 6.6, Germany) chromatography column as published earlier [16, 17]. CE and LA were qualitatively and quantitatively characterized by LC-MS and HPLC (Table 1).

#### 2.1.2. Identification of Compounds by LC-MS

Compounds were identified with the method described by Kucharska et al. [18] using the Acquity ultraperformance liquid chromatography (UPLC) system coupled with a quadruple time of flight (q-TOF) MS instrument (Waters Corp., USA) with an electrospray ionization (ESI) source. The Acquity BEH C18 column (100mm × 2.1mm i.d., 1.7 *μ*m; Waters Corp., USA) was used. The mobile phase was composed of solvents: A (2.0% aq. formic acid, *v*/*v*, Sigma-Aldrich, Germany) and B (100% acetonitrile, POCH, Poland). The instrument was operated in both the positive and negative ion modes, scanning *m*/*z* from 100 to 1500.

#### 2.1.3. Quantification of Compounds by HPLC-PDA

Iridoids and anthocyanins were assayed using the method described earlier [18] with an HPLC system equipped with the UltiMate 3000 model photodiode array detector (Dionex, Germany). The Cadenza Imtakt column CD-C18 (75 4.6 mm, 5 *μ*m) was used. The mobile phase was composed of solvents: A (4.5% aq. formic acid, *v*/*v*, Sigma-Aldrich, Germany) and B (100% acetonitrile, Sigma-Aldrich, Germany). Runs were monitored at wavelengths of 245 nm (iridoids and ellagic acid), 320 nm (phenolic acid), 360 nm (flavonols), and 520 nm (anthocyanins). Iridoids, phenolic acids, and anthocyanins were quantified as loganic acid, 5-caffeoylquinic acid, and cyanidin 3-*O*-glucoside, respectively. Flavonols were quantified as quercetin 3-*O*-glucoside and kaempferol 3-*O*-glucoside (Extrasynthese, France).

### 2.2. *In Vitro* Studies

#### 2.2.1. *E*. *coli* Strains

A prototype AIEC strain LF82 (O83:H1) isolated from a chronic ileal lesion of a patient with Crohn's disease, kindly provided by doctor Arlette Darfeuille-Michaud, Université d'Auvergne, France, and nonpathogenic *E*. *coli* strain K-12 C600 as a negative control were used in this study. *E*. *coli* strains were routinely cultured in Luria broth (LB) and subcultured on MacConkey agar (MCA) [19].

#### 2.2.2. Antimicrobial Assay

To evaluate the antibacterial activity of cornelian cherry iridoid-polyphenolic extract and loganic acid for both the *E*. *coli* LF82 and K-12 C600 strains, conventional microdilution assay based on the Clinical and Laboratory Standards Institute (CLSI) was used [20]. CE and LA at the concentrations of 256 mg/ml were serially twofold diluted in Mueller-Hinton broth (MHB) in a microtiter plate. Midlogarithmic phase (18 hours) *E*. *coli* culture in LB was adjusted to 0.5 McFarland standard turbidity in sterile normal saline and inoculated to the diluted CE and LA to the final density 5 × 10^5^ CFU/ml and incubated aerobically for 18 h at 35°C. Positive controls contain MHB with *E*. *coli* strains, and negative controls contain MHB with CE and LA, but no bacteria were included for every assay to determine adequate *E*. *coli* growth over the course of the incubation period and medium sterility, respectively. Additionally, gentamycin was used as a positive control. Afterward, the MIC and MBC values were determined. The lowest concentration of CE and LA that produced no visible growth (no turbidity) in comparison with control wells was considered the minimal inhibitory concentration (MIC). To determine the minimal bactericidal concentration (MBC) of samples of 50 *μ*l from the last, wells with visible bacterial growth and wells with no visible turbidity were inoculated onto nutrient agar and incubated aerobically overnight at 37°C. The highest dilution of CE and LA that yielded no single bacterial colony on the nutrient agar plates was considered an MBC value.

#### 2.2.3. Adherence Assay

All cell culture reagents and media were from Gibco (Thermo Fisher Scientific, Poland). Human intestinal epithelial Int407 cells (ATCC CCL-6™) were cultured in Dulbecco's modified Eagle's medium (DMEM) supplemented with heat-inactivated 10% fetal bovine serum (FBS) and antibiotic-antimycotic solution (AAS; penicillin 100 U, streptomycin 100 U, and amphotericin B 0.25 *μ*g per ml). For adherence assay, Int407 cells were seeded on 48-well plates at the density 2 × 10^5^ cells per well and cultured until 80% confluence. 24 h before assays, the cells were washed three times with phosphate-buffered saline (PBS; pH 7.2) and treated with serial twofold dilutions of cornelian cherry iridoid-polyphenolic extract and loganic acid in DMEM without antibiotics ranging from 2 to 10 mg/ml. Int407 epithelial cells in DMEM without antibiotics untreated with a water solution of CE and LA served as a negative control. The *in vitro* adherence assay to Int407 cells was performed according to Cravioto et al. [21]. *E*. *coli* strains cultured overnight at 37°C in LB, centrifuged, and resuspended in PBS (pH 7.4) to the optical density OD = 6 × 10^8^ colony-forming units per ml (CFU/ml) were used to infect Int407 cells untreated and treated with a water solution of CE and LA at a multiplicity of infection (MOI) 50 bacteria per one cell. After 3 h of incubation, Int407 cells were washed three times and lysed with 0.1% Triton X-100 in LB for 90 min to release cell-associated bacteria [22]. The 100 *μ*l aliquots of bacterial lysates were taken, and 10-fold dilutions were plated onto MacConkey agar plates to enumerate viable CFU. The adherence assay was carried out in three separate experiments.

#### 2.2.4. Yeast Agglutination Assay

To determine the effect of CE and LA on type 1 fimbriae of *E*. *coli*, the agglutination of yeasts was performed according to Korhonen [23]. Tested *E*. *coli* strains were grown for 48 h in a static tryptic soy broth at 37°C, harvested, and adjusted to OD_600_6 × 10^8^ CFU/ml. Agglutination was performed on glass slides by mixing 20 *μ*l *E*. *coli* with an equal volume of 2% baker's yeast suspension in water with and without an equal volume of CE and LA at the concentration of 128 mg/ml.

### 2.3. *In Vivo* Studies

#### 2.3.1. Ethical Considerations

The animal care and all experimental procedures were in accordance with the applicable international, national, and institutional guidelines and reviewed and approved by the Local Ethics Committee for Experiments on Animals in Wroclaw (No. 17/2017).

#### 2.3.2. Animals

Male Wistar rats (220-260 g) were purchased from the Animal Research Center at Wroclaw Medical University (Wrocław, Poland) and throughout the acclimatization and study periods kept in pairs in polypropylene cages with water *ad libitum*. Rats were housed in a controlled environment at a temperature of 21-24°C and humidity of 55-60% in 12 h light/dark cycle with free access to standard rodent chow (Agropol, Poland) except for the single procedure of deprivation.

#### 2.3.3. Experimental Design

The schedule of studied compound administration (16 days of pretreatment) was chosen in order to assess whether these compounds prevent occurrence or decrease colon damages induced by TNBS administration that resembles the most prevention of exacerbations of IBD after the remission period in humans. After 7 days of the handling period, rats were randomly assigned to 11 groups (7-8 rats in each) as follows: control group receiving distilled water intragastrically (IG) and once saline *per rectum* (PR), colitis (TNBS) group receiving distilled water IG and once TNBS solution PR, and 9 groups receiving IG: CE (20 or 100 mg/kg) or LA (10 or 50 mg/kg) or sulfasalazine (SA, 100 mg/kg) or CE (20 or 100 mg/kg) with SA (100 mg/kg) or LA (10 or 50 mg/kg) with SA (100 mg/kg) and once TNBS solution PR. Distilled water, CE, LA, and SA were given once daily by a gastric tube (4 ml/kg b.w.) for 16 consecutive days. Normal saline and TNBS solution were given rectally on the 15^th^ day of the experiment, after 24 h of food deprivation. Animals were observed and weighed daily. Forty-eight hours after induction of colitis, rats were sacrificed by cervical dislocation under deep pentobarbital anesthesia (200 mg/kg, Biowet, Poland). The distal 8 cm segment of the colon was excised from each rat, placed on an ice-cold plate, and longitudinally opened, cleaned, weighed, and subjected to macroscopic assessment. Afterward, samples were divided into three pieces. The first piece was fixed in 4% buffered formalin for histopathological examination. The second piece was used to prepare tissue homogenates. After centrifugation at 4000 rpm for 10 min at 4°C, the supernatants were transferred into new tubes and stored at -80°C for biochemical analyses. The third piece was used in gene expression analysis.

#### 2.3.4. Induction of Experimental Colitis

Experimental colitis was induced by 2,4,6-trinitrobenzenesulfonic acid (Sigma-Aldrich, Germany) according to the well-established colitis model originally described by Morris et al. [24]. Briefly, rats were anesthetized with ketamine (75 mg/kg, Biowet, Poland) and medetomidine (0.5 mg/kg, Orion Pharma, Poland) and positioned on their right side. Then, TNBS (50 mg/kg) dissolved in 50% ethanol (*v*/*v*, ChemPur, Poland) was infused into the colon via a polyethylene catheter (external diameter 2 mm) inserted into the lumen of the colon through the rectum with the tip positioned approximately 8 cm proximal to the anus. For the next 5 min, animals were kept in the Trendelenburg position to prevent leakage of the instilled solution.

#### 2.3.5. Assessment of the Body Weight and Colon Index

Throughout the experimental period, animals were weighed once daily. The difference in body weight between the 15^th^ and 17^th^ days of the experiment was determined. For each excised 8 cm long specimen of the colon, colon wet weight was measured. Then, the colon index was calculated as the ratio of colon wet weight to total body weight.

#### 2.3.6. Macro- and Microscopic Assessment of Colon Injury

Colon macroscopically visible damage was examined blindly, immediately after resection using a 0-5 scale, which takes into account the area of inflammation and the presence or absence of ulcers [25]. Then, colon sections were collected for the histological examinations. The material was fixed in 4% buffered formalin (ChemPur, Poland), embedded in paraffin, cut into 4 *μ*m thick slices using a rotary microtome (Sakura Accu-Cut SRM, Netherlands), and placed on glass slides. Preparations were stained using three methods: hematoxylin-eosin, Giemsa, and alcian blue for histological assessment of colonic damage, cell infiltration, and mucus content, respectively. Tissue sections were evaluated, and images were taken by standard light microscopy (Olympus CX41 microscope coupled to a DP20 camera, Olympus Soft Imaging Solutions GmbH–cell^D ver. 3.2, Olympus, Germany). Histological damage was assessed in a blinded fashion by the experienced pathologist using the scoring scales, based on the previously presented criteria [26]. In hematoxylin-eosin staining, the result was the sum of points (0-25) scored by evaluation of each criterion. In Giemsa staining, inflammatory cell infiltration was assessed using a 0-4-point scale. Specimens stained with alcian blue were assessed by description considering goblet cell number and size and amount of sialomucins.

#### 2.3.7. Cytokine Quantification by Enzyme-Linked Immunosorbent Assay (ELISA)

The concentrations of IL-23, IL-17, TNF-*α*, and chemerin were measured in obtained supernatants with quantitative enzyme immunoassay rat specific kits: Rat IL-23 ELISA Kit, Rat IL-17 ELISA Kit, Rat TNF-*α* ELISA Kit, and Rat Chemerin ELISA Kit, respectively (Diaclone, France) following the manufacturer's instructions. All concentrations were expressed as pg/ml.

#### 2.3.8. RNA Isolation and Quantitative Real-Time Polymerase Chain Reaction (qPCR)

Tissue specimens were soaked in RNAlater (Ambion Inc., USA) and stored at -80°C until RNA isolation. Tissue fragments up to 40 mg were homogenized using FastPrep-24 Homogenizer (MP Biomedical, USA) in lysis buffer (Thermo Fisher Scientific, USA) with the addition of *β*-mercaptoethanol (Sigma-Aldrich, USA). RNA was isolated using phenol-chloroform extraction and purified using the PureLink™ RNA Mini Kit (Thermo Fisher Scientific). Genomic DNA was removed by on-column treatment of RNA isolates with DNase (PureLink™ DNase Set, Invitrogen™, Thermo Fisher Scientific). The concentration of isolated RNA was quantified using NanoDrop 2000 (Thermo Fisher Scientific) with a concomitant evaluation of RNA purity (ratios of absorbance at 260, 280, and 230 nm). Subsequently, 1 *μ*g of RNA per reaction mixture (20 *μ*l) was reversely transcribed in a C1000 thermocycler (Bio-Rad, Poland) using the iScript™ cDNA Synthesis Kit (Bio-Rad, Poland) and the manufacturer's protocol. All samples were accompanied by matching negative transcription (“no-RT”) controls, devoid of reverse transcriptase, and subsequently tested to assure a lack of contamination with genomic DNA. The quantitative (real-time) PCR (qPCR) reactions were conducted using the CFX96 Real-Time PCR system (Bio-Rad) and SsoFast EvaGreen® Supermix (Bio-Rad). The following cycling conditions were applied: 30 sec activation at 95°C, 5 sec denaturation at 95°C, annealing/extension for 5 sec at 61°C, and 40 cycles, followed by a melting step (60-95°C with fluorescent reading every 0.5°C). The reaction mixture contained 2 *μ*l of cDNA (diluted 1 : 7.5), 10 *μ*l of 2x SsoFast EvaGreen® Supermix, 1 *μ*l of each 10 nM forward and reverse target-specific primer, and water up to 20 *μ*l. Primer specificities were tested by melting curve analysis and electrophoresis in high-resolution agarose (SeaKem LE agarose, Lonza, Switzerland) in TBE with SYBR Green (Lonza) detection. Primers' sequences are presented in Table 2. Primers were synthesized by Genomed (Poland). Prior expression analysis technical replicates were averaged. For each sample, a normalized relative quantity (NRQ) of a given gene transcript was calculated using qBasePlus version 2.4 software (Biogazelle BE, Belgium). The NRQ was calculated as 2^(ΔCq : differencebetweenthegeometricmeanofCqinthewholegroup–sampleCq) normalized to GAPDH, used as an internal control.

### 2.4. Statistical Analysis

All experimental data are presented as meanvalues ± standarddeviation(SD). Statistical differences between studied parameters were analyzed using one-way analysis of variance (ANOVA) and multiple comparisons with Tukey's *post hoc* test. All statistical analyses were performed with GraphPad Prism version 8.0 (GraphPad Software, USA) with statistical significance set at *p* value < 0.05.

## 3. Results

### 3.1. Cornelian Cherry Iridoid-Polyphenolic Extract but Not Loganic Acid Exerts Antibacterial Activity *In Vitro*

The MIC values of CE against nonpathogenic *E*. *coli* K-12 C600 and pathogenic *E*. *coli* LF82 were 0.125 mg/ml and 0.0625 mg/ml, respectively, whereas MBC values were higher than MIC values and were the same for both *E*. *coli* strains (16 mg/ml). MIC and MBC values of reference gentamycin against both the nonpathogenic and pathogenic strains were 0.25 *μ*g/ml and 0.5 *μ*g/ml, respectively. Antibacterial agents are usually regarded as bactericidal if their MBC is no more than four times the MIC value. Thus, CE showed bacteriostatic rather than bactericidal activity against both *E*. *coli* strains as MBC/MIC ratios for *E*. *coli* K-12 C600 and *E*. *coli* LF82 were 128 and 256, respectively. On the contrary, loganic acid showed no antibacterial activity with MIC and MBC values > 128mg/ml for both *E*. *coli* strains.

### 3.2. Cornelian Cherry Iridoid-Polyphenolic Extract but Not Loganic Acid Inhibits Adherence of Pathogenic *E*. *coli* Strain LF82 to Intestinal Epithelial Cells and *E*. *coli* Mannose-Sensitive Type 1 Fimbria Function *In Vitro*

**Figure 1:**
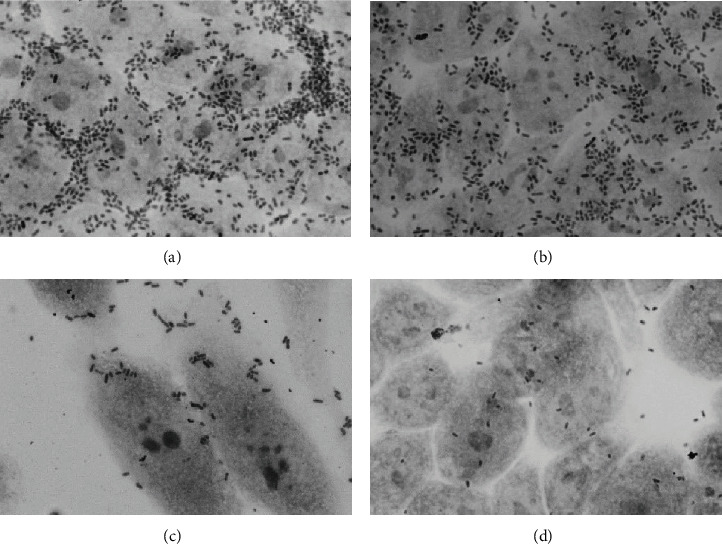
Adherence of *E*. *coli* strains to intestinal epithelial Int407 cells. *E*. *coli* strain LF82 in LB (a) and in LB with 2 mg/ml cornelian cherry iridoid-polyphenolic extract (c). *E*. *coli* strain K-12 C600 in LB (b) and in LB with 2 mg/kg cornelian cherry iridoid-polyphenolic extract (d). Magnification 100x.

**Figure 2:**
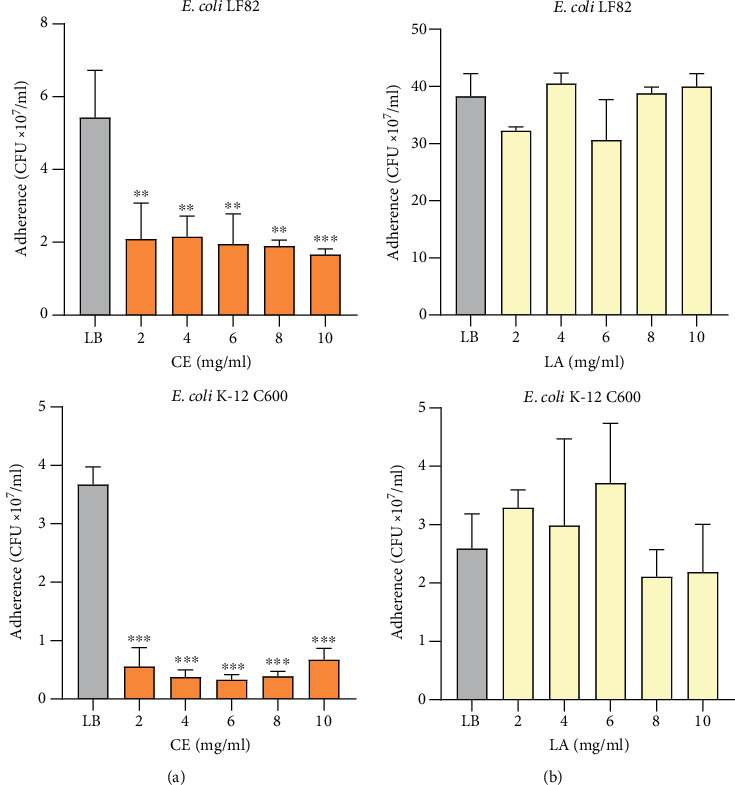
Adherence of *E*. *coli* LF82 and K-12 C600 to Int407 epithelial cells untreated (LB) and treated with different concentrations of cornelian cherry iridoid-polyphenolic extract (a) and loganic acid (b). Data are presented as meanvalues ± SD. Differences ^∗∗^*p* < 0.01 and ^∗∗∗^*p* < 0.001 were deemed statistically significant.

For *in vitro* adherence assay, increasing concentrations of CE and LA, ranging from 2 to 10 mg/ml, were used. Cornelian cherry iridoid-polyphenolic extract in all concentrations significantly inhibited adherence of both the pathogenic and nonpathogenic *E*. *coli* strains to intestinal epithelial cells (*p* < 0.01), and this action was independent of studied CE concentration (Figures 1 and 2). In contrast, loganic acid in all studied concentrations does not affect the adherence to IECs of both *E*. *coli* strains (Figure 2). Type 1 pili of *E*. *coli* are involved in their adherence to the IECs. Thus, the yeast cell agglutination assay was performed.

**Figure 3:**
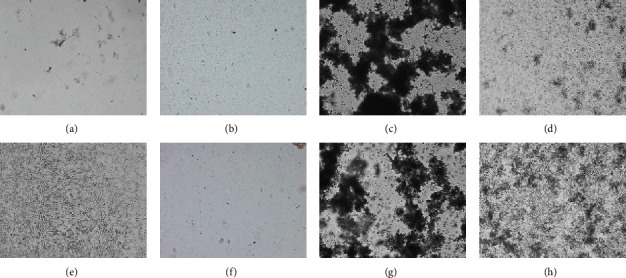
Yeast agglutination in the presence of cornelian cherry iridoid-polyphenolic extract. Negative controls were cornelian cherry iridoid-polyphenolic extract at the concentration of 128 mg/ml mixed with an equal volume of water (a), cornelian cherry iridoid-polyphenolic extract mixed with *E*. *coli* strain K-12 C600 (b), 2% yeast suspension with water (e), and *E*. *coli* strain LF82 mixed with cornelian cherry iridoid-polyphenolic extract (f). Positive controls were *E*. *coli* strain K-12 C600 mixed with 2% yeast suspension (c) and *E*. *coli* strain LF82 mixed with 2% yeast suspension (g). Experimental groups were *E*. *coli* strain K-12 C600 with 2% yeast suspension and cornelian cherry iridoid-polyphenolic extract (d) and *E*. *coli* strain LF82 mixed with 2% yeast suspension and cornelian cherry iridoid-polyphenolic extract (h). Magnification 20x.

Similar to the adherence test, only CE significantly reduced the agglutination of yeast cells by fimbriated *E*. *coli*. The control agglutination of yeast cells by both fimbriated *E*. *coli* strains was very strong and observed within a few seconds. Contrary, when *E*. *coli* strains were first mixed with an equal volume of CE, and then with yeast cells, the agglutination appeared after minutes and was much weaker compared to the agglutination of yeast cells without CE (Figure 3).

### 3.3. Cornelian Cherry Iridoid-Polyphenolic Extract but Not Loganic Acid Prevented the Body Weight Loss in Rats with TNBS-Induced Colitis

Significant body weight loss was noticed in the TNBS group in comparison to the control group (*p* < 0.05), suggesting that TNBS effectively induced colitis. The body weight loss was remarkably improved only in the group pretreated with CE at the high dose (100 mg/kg) when compared to the TNBS group (*p* < 0.001), and this action was more effective than that provided by sulfasalazine (*p* < 0.01). The body weight loss in other pretreated groups was repressed, but the effect was not considered statistically significant. No significant differences were observed between studied groups as regards the colon index. A merely insignificant increase in the colon index was noticed in the TNBS group compared to the healthy control rats (Table 3).

### 3.4. Cornelian Cherry Iridoid-Polyphenolic Extract but Not Loganic Acid Protected from Macro- and Microscopic Pathological Changes in Rats with TNBS-Induced Colitis

**Figure 4:**
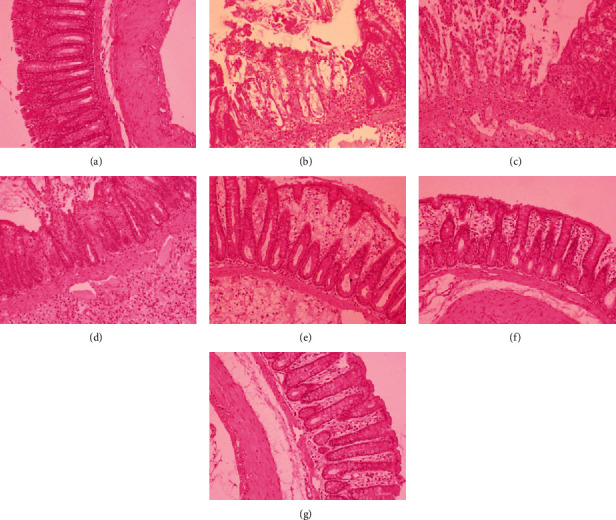
Microscopic appearance of colon tissues after hematoxylin-eosin staining demonstrated that cornelian cherry iridoid-polyphenolic extract reduces histological damage. Control group (a); group receiving only TNBS (b); groups receiving 20 or 100 mg/kg cornelian cherry extract with TNBS, respectively (c, d); group receiving 100 mg/kg sulfasalazine with TNBS (e); groups receiving 20 or 100 mg/kg cornelian cherry extract and 100 mg/kg sulfasalazine with TNBS, respectively (f, g). Magnification 100x.

**Figure 5:**
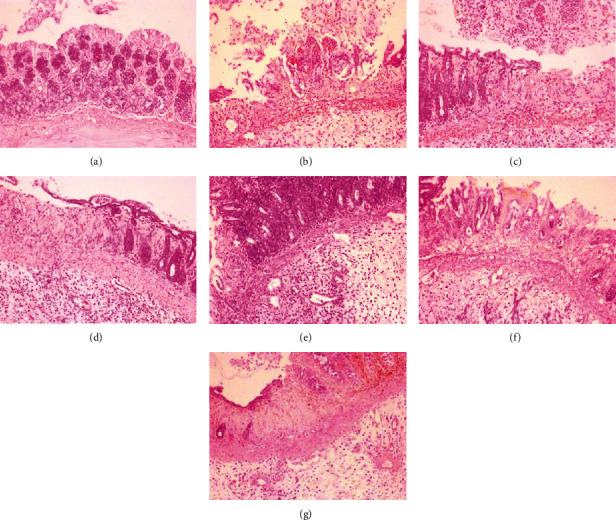
Microscopic appearance of colon tissues after Giemsa staining demonstrated that cornelian cherry iridoid-polyphenolic extract reduces inflammatory cell infiltration. Control group (a); group receiving only TNBS (b); groups receiving 20 or 100 mg/kg cornelian cherry extract with TNBS, respectively (c, d); group receiving 100 mg/kg sulfasalazine with TNBS (e); groups receiving 20 or 100 mg/kg cornelian cherry extract and 100 mg/kg sulfasalazine with TNBS, respectively (f, g). Magnification 100x.

In TNBS-induced colitis, macroscopic damages (performed on a 0-5-point scale) involved bowel wall thickening, hyperemia, and edema, and when compared to the control group, these changes were significant (*p* < 0.001). Epithelial damages, inflammatory cell infiltration, and mucosal architecture disorganization were defined as the three main categories with respective subordinate specific criteria described in detail in the legend of Table 4. In a microscopic examination, mucosal hyperemia with significant declines and ulcerations involving not only the epithelial layer but also all intestinal layers in some of the studied specimens was observed (Figure 4). Infiltration of inflammatory cells, including lymphocytes, macrophages, and neutrophils, ranging from mainly focal mucosal localization through the submucosa and the muscularis propria to transmural infiltrates in the most severe cases was found. Pronounced edema of the mucus and submucous membrane along with strong stimulation of the lymphatic apparatus was noticed. Major changes that originally apply to epithelial cell layers included crypt epithelial cell hyperplasia, the loss of goblet cells, cryptitis and crypt abscesses, and erosions. In specimens of TNBS-induced colitis, structural distortion of crypts and desquamated areas or loss of the epithelium was shown (Figures 4 and 5).

**Figure 6:**
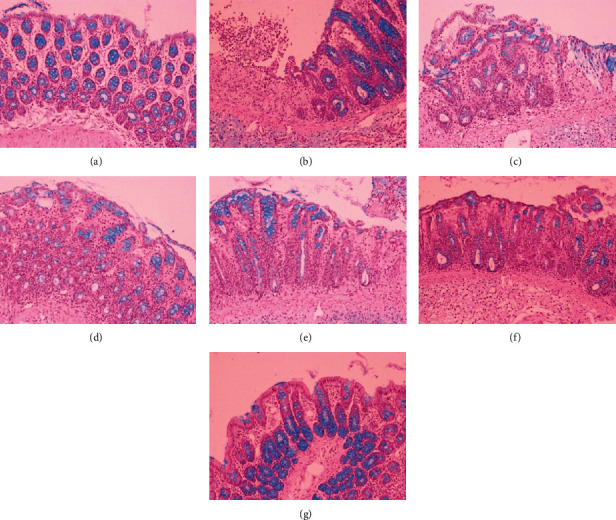
Microscopic appearance of colon tissues after alcian blue staining demonstrated that cornelian cherry iridoid-polyphenolic extract prevents loss of the mucus layer. Control group (a); group receiving only TNBS (b); groups receiving 20 or 100 mg/kg cornelian cherry extract with TNBS, respectively (c, d); group receiving 100 mg/kg sulfasalazine with TNBS (e); groups receiving 20 or 100 mg/kg cornelian cherry extract and 100 mg/kg sulfasalazine with TNBS, respectively (f, g). Magnification 100x.

Intestinal inflammation was linked with goblet cell depletion leading to the decline of the mucus layer of the epithelium. Colons in the TNBS group showed less regular structure of goblet cells, their smaller number, less frequent cell distribution in crypts and villi, and less mucus. At the regions of mucosal damage, goblet cells were damaged, and mucus was present extracellularly within the damaged and swollen submucosa. No goblet cells in the ulcer center, with only a small number of them on the ulceration periphery and normal number only in undamaged mucosa, were found (Figure 6).

Scoring of colonic tissue samples from each group indicates that pretreatment with cornelian cherry iridoid-polyphenolic extract at the high dose (100 mg/kg) or with sulfasalazine attenuated the colonic lesions induced by TNBS and thereby reduced macro- and microscopic damages contrasted to the TNBS group (*p* < 0.05 in all cases). Similarly, coadministration of CE at the dose of 100 mg/kg with sulfasalazine remarkably prevented the macro- and microscopic damages in comparison to the TNBS group (*p* < 0.01 in all cases), but the action of combined pretreatment was not greater than administration of each one alone (*p* = NS). In histological examination, rats pretreated with CE or SA alone or CE with SA showed explicit recovery of colon tissues with decreased extent and intensity of ulceration and reduced inflammatory cell infiltration (Figures 4 and 5). Only CE- or CE with SA-pretreated rats revealed an increased amount of goblet cells and restored epithelial cell layers. In these rats, goblet cells were characterized by an abundant amount of mucus, regular structure, almost equal size, and regular distribution in intestinal crypts in both the longitudinal and transverse sections (Figure 6).

### 3.5. Cornelian Cherry Iridoid-Polyphenolic Extract Counteracted Increased Colonic Levels of Proinflammatory Cytokines in Rats with TNBS-Induced Colitis

The concentrations of IL-23, IL-17, TNF-*α*, and chemerin were significantly increased by TNBS administration as compared to the control group (*p* < 0.01, *p* < 0.01, *p* < 0.001, and *p* < 0.01, respectively). Pretreatment with CE at the high dose (100 mg/kg) prevented from this increase and normalized all studied cytokine concentrations in colon tissues when compared with the group receiving only TNBS (*p* < 0.001, *p* < 0.01, *p* < 0.01, and *p* < 0.05, respectively), while the administration of CE at the low dose (20 mg/kg) prevented only from the IL-17 concentration increase (*p* < 0.05). Pretreatment with cornelian cherry iridoid-polyphenolic extract at the high dose counteracted increased IL-23 and chemerin levels more effectively than sulfasalazine (*p* < 0.01 and *p* < 0.05, respectively). Studied extract at the high dose given in combination with sulfasalazine also normalized concentrations of all studied cytokines as compared to the TNBS group (*p* < 0.05, *p* < 0.001, *p* < 0.001, and *p* < 0.05, respectively), and the effect on IL-17, TNF-*α*, and chemerin levels was even greater than after sulfasalazine in monotherapy (*p* < 0.05 in all cases). It indicates that cornelian cherry iridoid-polyphenolic extract not only strongly attenuated inflammatory response in rat colon tissues but also can act synergistically with sulfasalazine. Administration of loganic acid at the high or low dose alone or in combinations with sulfasalazine did not significantly improve the course of experimental colitis, considering almost all studied cytokines. Only pretreatment with LA at the high dose, together with sulfasalazine, significantly protected from the increase of the TNF-*α* concentration in comparison to the colitis group (*p* < 0.01), but the action of combined treatment was not greater than the administration of sulfasalazine alone (*p* = NS; Table 5).

### 3.6. Cornelian Cherry Iridoid-Polyphenolic Extract and Loganic Acid Regulated Muc2, TFF3, and STAT3 mRNA Expression in Rats with TNBS-Induced Colitis

**Figure 7:**
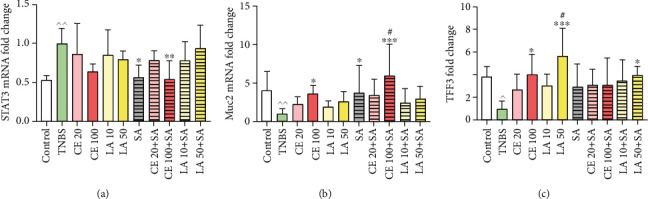
The impact of cornelian cherry iridoid-polyphenolic extract and loganic acid on the gene expression of transcription factor STAT3 (a), intestinal mucosal barrier protein Muc2 (b), and TFF3 (c) in experimental groups. Control = control group; TNBS = group receiving only TNBS; CE20, CE100 = groups receiving 20 or 100 mg/kg cornelian cherry extract with TNBS, respectively; LA10, LA50 = groups receiving 10 or 50 mg/kg loganic acid with TNBS, respectively; SA = group receiving 100 mg/kg sulfasalazine with TNBS; CE20+SA, CE100+SA = groups receiving 20 or 100 mg/kg cornelian cherry extract and 100 mg/kg sulfasalazine with TNBS, respectively; and LA10+SA, LA50+SA = groups receiving 10 or 50 mg/kg loganic acid and 100 mg/kg sulfasalazine with TNBS, respectively. Relative expression levels were analyzed by qPCR. Data are presented as meanvalues ± SD. Differences ^^^*p* < 0.05 vs. the control group; ^^^^*p* < 0.01 vs. the control group; ^∗^*p* < 0.05 vs. the TNBS group; ^∗∗^*p* < 0.01 vs. the TNBS group; ^∗∗∗^*p* < 0.001 vs. the TNBS group; and ^#^*p* < 0.05 vs. the sulfasalazine group were deemed statistically significant.

Induction of colitis caused a decrease in intestinal Muc2 and TFF3 mRNA levels and an increase in STAT3 mRNA expression as compared to the control group (*p* < 0.01, *p* < 0.05, and *p* < 0.01, respectively). Pretreatment with CE at the high dose (100 mg/kg) resulted in more than a 3.5-fold increase in the Muc2 mRNA level as compared to the TNBS group (*p* < 0.05), and this effect was close to that provided by sulfasalazine. Concomitantly administrated CE at the high dose and sulfasalazine elevated the Muc2 mRNA level near 6-fold in comparison to the colitis group (*p* < 0.001), and the effect of combined treatment was even greater than the administration of CE or sulfasalazine alone (*p* < 0.05 in both cases). Administration of CE at the high dose resulted in a 4-fold increase in the TFF3 mRNA level as compared to the TNBS group (*p* < 0.05) in contrast with SA, whose effect was not statistically significant (*p* = NS). Pretreatment with loganic acid at the high dose (50 mg/kg) elevated TFF3 mRNA expression as compared to TNBS-subjected rats (*p* < 0.001) in a more efficient way than with SA (*p* < 0.05). In contrast, for coadministration of LA at the high dose, SA was not more effective than administration of sulfasalazine alone (*p* = NS). Increased expression of STAT3 mRNA was inhibited only when the group receiving TNBS was pretreated with sulfasalazine (0.56-fold) or CE at the high dose together with sulfasalazine (0.54-fold) (*p* < 0.05 and *p* < 0.01, respectively), but the combined treatment gave no additional effect (*p* = NS; Figure 7).

## 4. Discussion

As stated in Introduction, the research was carried out to evaluate the impact of cornelian cherry iridoid-polyphenolic extract and loganic acid on AIEC strain growth and adhesion to intestinal epithelial cells as well as assessing the influence of CE and LA and the combination of each of these phytocompounds with sulfasalazine on intestinal inflammation in experimental colitis. Despite the accessibility of genetic and spontaneous models that mimic IBD, TNBS-induced colitis remains a valuable implement in the preclinical studying of various natural or chemical compounds in terms of their anti-inflammatory and immunomodulatory effects. Moreover, the TNBS model shares many biochemical and immunological features and symptoms with the disease in humans, especially with human CD, which has a predominant Th1 and Th17 profile [11, 27].

Impaired mucosal epithelial barrier integrity results in a thinner mucus layer due to goblet cell depletion and is associated with both the pathogenesis of IBD and experimental TNBS-induced colitis [27]. The defective mucosal barrier leads to interactions between the luminal contents, such as intestinal microbiota and the underlying immune system triggering the exacerbated intestinal immune response [11]. Microbiota and microbial products can modulate mucin synthesis and secretion by direct activation of diverse signaling cascades or via factors produced by epithelial and lamina propria cells. Normally, the mucus layer is in equilibrium between synthesis and decay by the colonic microbiota. Pathogens circumvent the protective function of the mucus layer by developing specific mechanisms to subvert and penetrate the mucus barrier [28]. AIEC strain LF82 is a good case in point. First, AIEC overcomes the mucus layer owing to Vat-AIEC protease which causes the degradation of mucins and decreases mucus viscosity [29], then strongly adheres to and invades intestinal epithelial cells, and colonizes gut mucosa by binding type 1 pilus mannose-specific adhesins (FimH) to the host carcinoembryonic antigen-related cell adhesion molecules (CEACAM6) [6, 30].

Moreover, AIEC is able to survive and replicate within macrophages, inducing the secretion of TNF-*α* and promoting granuloma formation [7]. The strain LF82 can colonize intestinal mucosa in the absence of overt disease yet able to induce intestinal inflammation in genetically predisposed individuals [31]. AIEC strains are primarily implicated in the ileal form of CD [19]. However, more recent studies have shown an increased prevalence of AIEC strains in the mucosa not only in CD but also in UC patients compared to healthy subjects [32]. Thus, the bacteriostatic and antiadhesive activities of CE against AIEC strains indicated in this paper suggest a possible therapeutic use of this extract in the treatment of AIEC-colonized IBD patients. In a previous study, Milenković-Andjelković et al. [33] showed that cornelian cherry fruit extract exerted antimicrobial activity against *E*. *coli* bacteria with MIC and MBC values 125 and 250 *μ*g/ml, respectively. The MIC value for CE obtained in our study against nonpathogenic *E*. *coli* strain was the same as in the study of Milenković, whereas MIC against adherent-invasive *E*. *coli* was 62.5 *μ*g/ml. It may give some potential benefits in the prevention of physiological microbiome dysfunction using CE with the action predominantly against pathological bacteria. Differences in MBC values in both studies are difficult to explain and may be caused by, e.g., different solvents and conditions used to prepare extracts. Antimicrobial activity of CE can be attributed to its high phenolic compound content, which has been reported to disintegrate the outer membrane of Gram-negative bacteria, releasing LPS and increasing the permeability of the cytoplasmic ATP [34, 35]. Adhesion of bacteria to host cells is a prerequisite for successful microbial colonization [35, 36]. The antiadhesive effect of CE, similar to other polyphenol-rich sources such as cranberry fruits, most probably was associated with the ability of its compounds to interact with FimH adhesins located at the distal tip of pili and constitutes an important adhesive protein promoting *E*. *coli* binding to the host cells [35–37]. The interaction between AIEC type 1 pili and oligomannose glycans on the surface of intestinal epithelial cells plays a key role in enabling AIEC to become established on the intestinal epithelium [30]. Type 1 pili are responsible for mannose-sensitive yeast cell agglutination as D-mannose, and its analogs inhibit this effect [38]. The ability of CE to reduce yeast cell agglutination via type 1 piliated *E*. *coli* strongly suggests that the compounds of cornelian cherry iridoid-polyphenolic extract interact with the adhesins. The therapeutic rationale for FimH antagonists in IBD is to prevent bacteria binding to IECs and wash them out of the gut without disruption of the intestinal microbiota. This paper fits in with the trend of searching adhesin antagonists as a treatment approach for IBD. Some efforts have been recently made to identify small-molecule FimH antagonists that target the mannose-binding lectin domain of FimH, inhibiting its function and preventing AIEC binding to mannosylated host cells [39].

In addition to bacteriostatic and antiadhesive properties of the cornelian cherry iridoid-polyphenolic extract, the results found in this study revealed that CE enhanced mucosal epithelial barrier restoration and protected the goblet cells and their content, such as Muc2 and TFF3. Muc2 is the predominant colonic mucus layer mucin that provides essential defense against endo- and exogenous irritants and bacterial adhesion to underlying epithelial cells [28]. The importance of Muc2 for maintenance of mucosal integrity is emphasized in Muc2-deficient mice, which developed spontaneous colitis and were susceptible to experimental colitis induced by dextran sodium sulfate (DSS) [40]. TFF3 interacts with Muc2 to reinforce the structural integrity of the intestinal barrier. Moreover, TFF3 is indispensable to restore the continuity of the epithelium and regeneration of mucosal integrity after injury. The compelling role of TFF3 in resealing the intestinal epithelial barrier is to enable the migration of viable epithelial cells from the injury edge to the denuded area, so-called restitution [41]. Thus, an increase in Muc2 and TFF3 expression should contribute to improving mucosal epithelial barrier architecture and function. This kind of action has been demonstrated in this study, and as far as we know, this work is the first to evaluate the effect of cornelian cherry iridoid-polyphenolic extract and loganic acid and concomitant administration of these phytocompounds with sulfasalazine in rat experimental colitis. Cornelian cherry iridoid-polyphenolic extract restored the expression of proteins involved in epithelial integrity, such as Muc2 and TFF3, which were shown at the mRNA level. In turn, sulfasalazine as a standard drug in IBD treatment was able to increase only Muc2 expression. This is consistent with findings reported by other authors who have shown that sulfasalazine given prior or after induction of inflammation by TNBS increased only Muc2 mRNA expression, whereas polyphenol-rich plant-derived extracts augmented both Muc2 and TFF3 expression [42, 43]. Besides increasing expression of Muc2 and TFF3, polyphenols have also been stated to be able to cross-link mucin, enhancing the viscosity and elasticity of the mucus layer [44], thereby stabilizing the intestinal mucus layer [45]. It is noteworthy that concomitantly administrated CE and SA exerted greater effect than each one alone in regard to Muc2 expression, which suggests that CE acts synergistically with SA. The content of mucin in the mucosa was confirmed by histological evaluation with alcian blue staining which showed the restored mucosal epithelial layer, increased number and size of goblet cells and amount of acid glycoproteins (mainly sialomucins), and improved adherents and tight junctions leading to the appropriate cover of the mucus layer over the intestinal epithelium after CE and CE with SA treatment prior to colitis induction. A more pronounced protective effect exhibited by CE with SA can be explained by the fact that Muc2 and TFF3 together act more effectively in protecting epithelial cells when compared with either one alone [46], which indicates their joint effect in mucosal protection.

Interestingly, the expression of TFF3 mRNA was the most upregulated after pretreatment with loganic acid at the high dose (50 mg/kg). The action of LA was significantly greater than the effect of sulfasalazine. It may suggest that loganic acid is a crucial ingredient required to improve TFF3 but not Muc2 expression. Quite possibly, other than LA ingredients from cornelian cherry extract are connected with the Muc2 expression increase and provide synergistic action between SA and CE, i.e., ellagic acid. Rosillo et al. [47] indicated that ellagic acid enhanced mucus production by goblet cells in the colon mucosa in TBNS-induced colitis.

Restoration of the mucosal epithelial barrier and thereupon separation of an antigen-rich gut lumen from the lamina propria immune system can contribute to reduce exaggerated antigenic stimulation of nonspecific and specific immune responses and can lead to the inhibition of intestinal wall inflammation characterized by excessive production of proinflammatory cytokines [28]. It is well known that TNF-*α* is implicated in the initiation and perpetuation of intestinal inflammation in IBD [48]. Recently, an overwhelming number of clinical and experimental observations showed the IL-23/IL-17 axis as an essential mediator involved in IBD [4, 49–51]. IL-23, produced by activated monocytes, macrophages, and dendritic cells, acts on the innate immune system cells, contributes to the inflammatory cytokine production and tissue inflammation, and promotes the expansion and maintenance of Th17 cells, which secrete the proinflammatory cytokine IL-17 involved in neutrophil activation and proinflammatory mediator production from different cells [4, 49]. IL-17 is produced predominantly by Th17 cells but also by *γδ* T cells and innate lymphoid cells (ILCs) [50]. Th17 differentiation from naive T cells and IL-17 expression firmly depend on transcription factor STAT3 and cytokines such as IL-1*β* and IL-6 [52]. Contrary, IL-23 is not strictly required for Th17 differentiation but plays a crucial role in maintenance, expansion, and pathogenicity of the Th17 cell phenotype and facilitates the production of IL-17 from Th17 and ILCs, but not *γδ* T cells [51]. Moreover, IL-23 stimulates Th17 and ILCs to produce TNF-*α*, granulocyte-macrophage colony-stimulating factor (GM-CSF), and several chemokines leading to immune response induction and inflammatory cell infiltration [49]. Another inflammatory mediator, chemerin, released by colonic epithelial cells, is an attractant for immune cells. Likewise, chemerin contributes to tissue macrophage recruitment, enhances IL-6 and TNF-*α* secretion in colonic epithelial cells, and suppresses the anti-inflammatory M2 macrophage response, which is most likely associated with the higher release of inflammatory cytokines [53]. TNF-*α*, IL-23, IL-17, and chemerin levels and gene expression of STAT3 are significantly elevated and positively correlated with the severity of both IBD and experimental colitis [4, 27, 48, 54]. Recently, therapeutic compounds that antagonize inflammatory mediators have been introduced into clinical practice. Monoclonal antibodies against TNF-*α* are now used and have been proven effective for the treatment of IBD patients [48, 55]. At present, several antibodies acting via IL-23/IL-17 pathway inhibition have been developed and examined in several preclinical and clinical studies [55]. Although phytocompounds studied in this work do not belong to monoclonal antibodies, they are also targeted at blocking proinflammatory cytokines. We have indicated that cornelian cherry iridoid-polyphenolic extract at the high dose (100 mg/kg) significantly reversed the increased colonic level of all studied cytokines but not STAT3 expression caused by TNBS whereas sulfasalazine normalized TNF-*α* and STAT3 expression. Similarly, results described by da Silva et al. indicating that SA decreased gene expression of STAT3 and the colonic level of TNF-*α* [56]. While our study was in progress, Süntar et al. [57] reported that extract of cornelian cherry at a very high dose (400 mg/kg) administered for 14 days after TNBS-induced colitis decreased TNF-*α* and IL-1*β* levels and the effect was close to that of sulfasalazine given at the same dose as in our study (100 mg/kg). It is worth mentioning that in our study, all tested compounds were used as prevention before colitis induction, while in Süntar et al.'s study, cornelian cherry extract was given after TNBS administration as a colitis treatment.

Phenolic compounds are known for their potent ability to modulate crucial inflammatory signaling pathways and gene regulation of several inflammatory enzymes and cytokines [58, 59]. Although several studies demonstrated that polyphenols exhibited a strong attenuating effect against colitis, there are only a few papers concerning the effect of polyphenols on the IL-23/IL-17 axis and STAT3 expression in experimental colitis. Extract from *Terminalia catappa* containing ellagic acid—a phenolic compound present also in CE—reversed increased IL-23 colonic expression [60]. Cocoa phenolic compounds exhibited an anti-inflammatory effect decreasing the expression of TNF-*α*, IL-17, and STAT3 in colonic tissues during DSS colitis [61]. Apple polyphenols ameliorated DSS colitis and dampened the expression of proinflammatory transcripts, such as IL-6, IL-17, IL-22, and TNF-*α* [62]. The impact of polyphenols containing plant extracts [42, 43, 63, 64] or pure phenolic compounds [65, 66] on TNF-*α* and IL-17 colonic levels was studied by some other research studies. Furthermore, it is noteworthy that in TNBS-subjected rats, one of the polyphenol groups, anthocyanins, especially pelargonidin, not only reversed intestinal inflammation and increased expression of TNF-*α* and IL-6 but also promoted the anti-inflammatory M2 macrophage expansion, which is inhibited by chemerin [67]. We did not find any previous studies investigating the impact of polyphenolic compounds, including cornelian cherry extract on chemerin tissue levels during TNBS colitis. To our best knowledge, our study is the first to evaluate the effect of concomitantly administered cornelian cherry iridoid-polyphenolic extract and sulfasalazine in experimental colitis in rats, indicating that such combined pretreatment prevented not only the increase in colonic cytokine levels but also the increase in TNBS-induced STAT3 expression. This combined therapy is even more effective than monotherapy with SA in regard to IL-17, TNF-*α*, and chemerin expression. The strong cytokine level normalization seems to be a crucial anti-inflammatory mechanism common to CE and SA, but the additional greater ability of CE and SA coadministration to decrease colonic cytokine levels may show the synergistic anti-inflammatory action between these compounds. It should be pointed out that besides the high efficacy of blocking IL-23 (e.g., by ustekinumab) as well as the important role of the IL-23/IL-17 axis in IBD pathogenesis and severity [4, 49–51, 68], the IL-17 neutralizing agent, secukinumab, is ineffective in CD patients in RCT [69]. A possible explanation for this negative result of IL-17 blockade may be complex molecular pathways underlying IBD with the same cytokine possessing both the protective and pathogenic effects on mucosal inflammation. As demonstrated by Lee et al. [50], IL-17 can be produced by multiple adaptive and innate cell populations. The source of gut-protective IL-17 was IL-23-independent *γδ* T cells as opposed to gut-pathogenic IL-17 produced by IL-23-dependent Th17 and ILCs. We believe that the studied extract decreasing the IL-23 level minimizes tissue inflammation, Th17, and ILC activation, whereas leaving the protective IL-17 from IL-23-independent *γδ* T cells unharmed. Further work needs to be carried out to establish whether CE reduces IL-17 production by *γδ* cells or Th17 and ILCs.

In addition to polyphenolic compounds studied, cornelian cherry extract contains a considerable amount of iridoids. It has been reported that an iridoid-rich fraction of *Syringae folium* extract possessed an anti-inflammatory effect against TNBS-induced colitis in rats through inhibiting proinflammatory cytokines (TNF-*α*, IL-6) by downregulating the expression of NF-*κ*B [70]. *Cornus officinalis* and its isolated iridoids, such as loganin and cornuside, showed anti-inflammatory effects in both the *in vitro* [71–73] and *in vivo* models [74, 75]. Sozański et al. [13] demonstrated that loganic acid, iridoid isolated from cornelian cherry fruits, exerted anti-inflammatory effects decreasing TNF-*α* and IL-6 concentrations during atherosclerosis. The authors also concluded that the main anti-inflammatory effects of the cornelian cherry fruit should be attributed to its iridoid fraction. Therefore, encouraged by these results, we decided to study the effect of not only the whole cornelian cherry extract but also the isolated loganic acid in the rat experimental colitis model. Based on the findings of the aforementioned authors, strong anti-inflammatory loganic acid activity was expected in the currently undertaken study [13, 70–75]. However, our results turned out to be surprising. Contrary to expectations, no activity of loganic acid in regard to inflammatory mediators was demonstrated in the TNBS-induced colitis model except for the TNF-*α* level decrease by combined LA and SA administration. This may indicate that loganic acid affects other proinflammatory signaling pathways not involved in IBD pathogenesis. Perhaps other iridoids present in cornelian cherry extract, e.g., cornuside, act as anti-inflammatory in colitis [71, 76]. We hypothesize that discrepancy between anti-inflammatory LA actions reported in the paper of Sozański et al. [13] and the current study may result from LA pharmacokinetics and its metabolism. Iridoids are considered well-absorbed compounds [77], which may be the reason for their low concentration in the inflamed colon. On the contrary, polyphenols, as particles of high molecular weight, have low gastrointestinal absorption but may exert a significant local effect in TNBS-induced colitis. It is estimated that approximately 90–95% of total dietary polyphenols reach the colon unabsorbed. Unabsorbed polyphenols are able to interact with the intestinal microbes and gut epithelium and exert their functions within the gut [78]. It cannot be excluded that alterations in the microbiome after TNBS can lead to the formation of inactive metabolites of iridoids resulting in poor anti-inflammatory activity in colitis. It, for sure, requires further research to thoroughly study the pharmacokinetics of loganic acid and microbiome in TNBS-induced colitis.

## 5. Conclusions

The results we found in this study point to the conclusion that cornelian cherry iridoid-polyphenolic extract exerted a protective effect against TNBS-induced colitis in rats via restoration of the damaged mucosal epithelial barrier and attenuation of the intestinal inflammatory response, presumably due to its polyphenolic content. Cornelian cherry iridoid-polyphenolic extract given concomitantly with sulfasalazine counteracted colitis in a more effective way than sulfasalazine alone, indicating a synergistic interaction between CE and SA. The multidirectional effect of CE seems to be beneficial when considering this extract as a candidate for adjuvant IBD therapy. CE could be coadministrated with any drug regardless of its mechanism. Iridoid-polyphenolic extract from the little-known, edible European fruit used in traditional folk medicine may constitute a promising source of phytocompounds to IBD therapy targeted at reinforcement of the mucosal epithelial barrier, modulation of the production of inflammatory mediators, and antiadhesive action. Further studies are needed to confirm that cornelian cherry iridoid-polyphenolic extract could constitute a preventive strategy or adjuvant treatment for patients suffering from IBD and whether an individual compound or rather a complex extract is more potent and promising in IBD attenuation.

## Figures and Tables

**Table 1:** Identification and the content (mg/100 g dry mass) of the main compounds of extract (CE) and loganic acid (LA) fraction from cornelian cherry fruits by LC-MS and HPLC.

Compound	[*M* − *H*]^−^/[*M* + *H*]^+^ (other ions) (*m*/*z*)	Content (mg/100 g dry mass)	
		Extract (CE)	Loganic acid (LA) fraction
Iridoids			
Loganic acid	375 (213)	10870.20 ± 15.86	69036.49 ± 1056.58
Cornuside 1	541 (169)	1549.28 ± 3.43	nd
Total iridoids		12419.48	69036.49
Phenolic acids			
Caffeoylhexoside	341 (179)	120.28 ± 3.00	nd
*p*-Coumaroilquinic acid 1	337 (163)	33.91 ± 1.40	nd
Caffeoylquinic acid	353 (191)	471.45 ± 1.42	127.77 ± 0.00
*p*-Coumaric acid	163	12.40 ± 0.21	nd
*p*-Coumaroilquinic acid 2	337 (191/163)	279.91 ± 3.50	261.85 ± 0.00
*p*-Coumaroilquinic acid 3	337 (191/163)	20.91 ± 1.88	nd
Ellagic acid	301	124.21 ± 1.64	nd
Total phenolic acids		1063.07	389.62
Anthocyanins			
Delphinidin 3-*O*-galactoside	463+ (303+)	44.06 ± 1.77	nd
Cyanidin 3-*O*-galactoside	449+ (287+)	809.70 ± 0.55	nd
Cyanidin 3-*O*-robinobioside	595+ (287+)	369.94 ± 1.41	nd
Pelargonidin 3-*O*-galactoside	433+ (271+)	1542.22 ± 0.84	nd
Pelargonidin 3-*O*-robinobioside	579+ (271+)	297.18 ± 0.44	nd
Cyanidin	287+	222.98 ± 31.61	nd
Pelargonidin	271+	396.55 ± 67.49	nd
Total anthocyanins		3682.63	
Flavonols			
Quercetin 3-*O*-glucuronide	477 (301)	306.28 ± 22.62	nd
Quercetin 3-*O*-glucoside	463 (301)	31.64 ± 1.58	nd
Kaempferol 3-*O*-galactoside	447 (285)	215.45 ± 2.61	nd
Kaempferol 3-*O*-glucuronide	461 (285)	35.94 ± 1.38	nd
Total flavonols		589.30	

Values are means ± SD. nd: not detected.

**Table 2:** Primers' sequences.

Symbol	Gene name	Accession no.	Primer sequence 5′ ⟶ 3′	Amp. size (bp)
*Gapdh*	Glyceraldehyde-3-phosphate dehydrogenase	NM_017008.4	F: tgactctacccacggcaagttcaa	141
			R: acgacatactcagcaccagcatca	
*Tff3*	Trefoil factor 3	NM_013042.2	F: taaccctgctgctggtcctg	195
			R: gtttgaagcaccagggcaca	
*Stat3*	Signal transducer and activator of transcription 3	NM_012747.2	F: cgccttggattgagagccaagat	112
			R: aggaatcggctatactgctggt	
*Muc2*	Mucin 2	XM_017604244.1	F: accaccattaccaccacctcag	119
			R: cgatcaccaccattgccattg	

Primer sequences were retrieved from the literature and validated *in silico* by BLAST analysis. Forward and reverse primer sequences are denoted by “F” and “R,” respectively. Amp.: amplicon; bp: base pairs.

**Table 3:** The impact of cornelian cherry iridoid-polyphenolic extract and loganic acid on the body weight loss and colon index in experimental groups. Control = control group; TNBS = group receiving only TNBS; CE20, CE100 = groups receiving 20 or 100 mg/kg cornelian cherry extract with TNBS, respectively; LA10, LA50 = groups receiving 10 or 50 mg/kg loganic acid with TNBS, respectively; SA = group receiving 100 mg/kg sulfasalazine with TNBS; CE20+SA, CE100+SA = groups receiving 20 or 100 mg/kg cornelian cherry extract and 100 mg/kg sulfasalazine with TNBS, respectively; and LA10+SA, LA50+SA = groups receiving 10 or 50 mg/kg loganic acid and 100 mg/kg sulfasalazine with TNBS, respectively. Data are presented as meanvalues ± SD. Differences ^^^*p* < 0.05 vs. the control group; ^∗∗∗^*p* < 0.001 vs. the TNBS group; and ^##^*p* < 0.01 vs. the sulfasalazine group were deemed statistically significant.

Group	Body weight loss (g)	Colon index
Control	14.71 ± 5.16	0.005 ± 0.001
TNBS	−5.13 ± 4.88^^^	0.009 ± 0.003
CE20	6.13 ± 13.24	0.007 ± 0.003
CE100	22.43 ± 6.60^∗∗∗^^##^	0.006 ± 0.002
LA10	3.86 ± 6.08	0.007 ± 0.002
LA50	7.25 ± 5.26	0.008 ± 0.002
SA	−1.13 ± 20.04	0.006 ± 0.003
CE20+SA	3.75 ± 12.96	0.007 ± 0.001
CE100+SA	3.86 ± 8.67	0.006 ± 0.001
LA10+SA	1.63 ± 9.02	0.008 ± 0.001
LA50+SA	6.75 ± 5.37	0.007 ± 0.002

**Table 4:** The impact of cornelian cherry iridoid-polyphenolic extract and loganic acid on macroscopic damage of colon tissues and microscopic damage of colon tissues in HE and Giemsa staining in experimental groups. Control = control group; TNBS = group receiving only TNBS; CE20, CE100 = groups receiving 20 or 100 mg/kg cornelian cherry extract with TNBS, respectively; LA10, LA50 = groups receiving 10 or 50 mg/kg loganic acid with TNBS, respectively; SA = group receiving 100 mg/kg sulfasalazine with TNBS; CE20+SA, CE100+SA = groups receiving 20 or 100 mg/kg cornelian cherry extract and 100 mg/kg sulfasalazine with TNBS, respectively; and LA10+SA, LA50+SA = groups receiving 10 or 50 mg/kg loganic acid and 100 mg/kg sulfasalazine with TNBS, respectively. Scoring scale of macroscopic evaluation of colonic tissue damage: 0 = no damage; 1 = hyperemia, no ulcers; 2 = linear ulcer with no significant inflammation; 3 = linear ulcer with inflammation at one site; 4 = two or more sites of ulceration or inflammation and ulceration or inflammation extending <1 cm; and 5 = two or more major sites of ulceration or inflammation extending >1 cm along the length of the colon. Microscopic evaluation of colonic tissue damage in HE staining: in the mucosal epithelium and lamina propria: ulceration (0–4), mononuclear cell infiltration (0–3), and polymorphonuclear cell infiltration (0–3); in the submucosa: edema (0–3), mononuclear cell infiltration (0–3), and polymorphonuclear cell infiltration (0–3); and in the muscular layer: mononuclear cell infiltration (0–3) and polymorphonuclear cell infiltration (0–3). Scoring scale: 0 = none; 1 = mild; 2 = moderate; 3 = severe; and 4 = full-thickness; maximum score: 25. Microscopic evaluation of colonic tissue damage in Giemsa staining: 0 = no changes; 1 = few inflammatory cells in the lamina propria; 2 = increased number of inflammatory cells including neutrophils in the lamina propria; 3 = accumulation of inflammatory cells reaching the submucosa; and 4 = transmural infiltration of inflammatory cells. Data are presented as meanvalues ± SD. Differences ^^^^*p* < 0.01 vs. the control group; ^^^^^*p* < 0.001 vs. the control group; ^∗^*p* < 0.05 vs. the TNBS group; and ^∗∗^*p* < 0.01 vs. the TNBS group were deemed statistically significant.

Group	Macroscopic damage score (0-5 points)	Microscopic damage score, HE staining (0-25 points)	Microscopic damage score, Giemsa staining (0-4 points)
Control	0	0	0
TNBS	3.38 ± 1.22^^^^^	12.88 ± 5.28^^^^^	2.50 ± 0.75^^^^
CE20	2.06 ± 1.12	6.13 ± 3.36	1.75 ± 1.55
CE100	1.29 ± 0.70^∗∗^	4.71 ± 2.49^∗^	0.64 ± 1.41
LA10	2.38 ± 0.79	8.13 ± 7.57	2.13 ± 1.28
LA50	2.13 ± 1.03	6.25 ± 6.54	1.50 ± 0.89
SA	1.63 ± 1.22^∗^	5.00 ± 3.55^∗^	1.25 ± 1.11
CE20+SA	2.31 ± 0.92	7.50 ± 3.51	1.75 ± 0.92
CE100+SA	1.43 ± 0.67^∗∗^	2.57 ± 2.07^∗∗^	1.29 ± 1.28
LA10+SA	2.06 ± 0.56	7.00 ± 6.41	1.38 ± 0.89
LA50+SA	2.13 ± 0.92	6.63 ± 4.60	1.25 ± 0.97

**Table 5:** The impact of cornelian cherry iridoid-polyphenolic extract and loganic acid on IL-23, IL-17, TNF-*α*, and chemerin concentrations in colon tissues in experimental groups. Control = control group; TNBS = group receiving only TNBS; CE20, CE100 = groups receiving 20 or 100 mg/kg cornelian cherry extract with TNBS, respectively; LA10, LA50 = groups receiving 10 or 50 mg/kg loganic acid with TNBS, respectively; SA = group receiving 100 mg/kg sulfasalazine with TNBS; CE20+SA, CE100+SA = groups receiving 20 or 100 mg/kg cornelian cherry extract and 100 mg/kg sulfasalazine with TNBS, respectively; and LA10+SA, LA50+SA = groups receiving 10 or 50 mg/kg loganic acid and 100 mg/kg sulfasalazine with TNBS, respectively. Data are presented as meanvalues ± SD. Differences ^^^^*p* < 0.01 vs. the control group; ^^^^^*p* < 0.001 vs. the control group; ^∗^*p* < 0.05 vs. the TNBS group; ^∗∗^*p* < 0.01 vs. the TNBS group; ^∗∗∗^*p* < 0.001 vs. the TNBS group; ^#^*p* < 0.05 vs. the sulfasalazine group; and ^##^*p* < 0.01 vs. the sulfasalazine group were deemed statistically significant.

Group	IL-23 (pg/ml)	IL-17 (pg/ml)	TNF-*α* (pg/ml)	Chemerin (pg/ml)
Control	325.3 ± 110.6	55.61 ± 25.66	155.2 ± 84.07	440.1 ± 177.9
TNBS	591.5 ± 149.5^^^^	97.88 ± 6.28^^^^	304.3 ± 64.21^^^^^	687.6 ± 108.7^^^^
CE20	405.4 ± 214.4	61.63 ± 14.22^∗^	200.7 ± 75.64	588.8 ± 177.3
CE100	301.3 ± 115.8^∗∗∗^^##^	58.07 ± 34.14^∗∗^	167.2 ± 21.28^∗∗^	455.5 ± 136.0^∗^^#^
LA10	541.6 ± 116.7	73.30 ± 16.30	212.4 ± 53.69	628.7 ± 123.3
LA50	492.9 ± 104.7	83.28 ± 16.26	197.6 ± 84.94	539.1 ± 106.5
SA	548.7 ± 131.9	74.00 ± 25.55	201.0 ± 67.18^∗^	665.9 ± 119.4
CE20+SA	408.5 ± 80.75	60.82 ± 16.48^∗∗^	99.67 ± 20.99^∗∗∗^	582.7 ± 46.95
CE100+SA	354.8 ± 57.49^∗^	40.33 ± 9.93^∗∗∗^^#^	83.62 ± 19.00^∗∗∗^^#^	458.3 ± 74.65^∗^^#^
LA10+SA	420.4 ± 107.9	73.39 ± 9.49	208.6 ± 77.08	664.6 ± 93.43
LA50+SA	391.6 ± 88.95	76.01 ± 11.61	162.8 ± 58.45^∗∗^	575.6 ± 48.8

## Data Availability

The data underlying this article will be shared on request to the corresponding author.

## Abbreviations

CE: Cornelian cherry iridoid-polyphenolic extract
LA: Loganic acid.

## Additional Points

*Conference Presentation*. Szandruk-Bender M., Sozański T., Rutkowska M., Merwid-Ląd A., Kwiatkowska J., Kucharska AZ., Piórecki N., Szeląg A. Effect of cornelian cherry iridoid-polyphenolic fraction and loganic acid on IL-23/IL-17 expression in colitis model in rats. 20th International Congress of the Polish Pharmacological Society, Lublin, Poland, 5-7 June 2019; 134-135.
